# etymologia: Leishmaniasis [lēsh-ma′-ne-ә-sis]

**DOI:** 10.3201/eid1404.070666

**Published:** 2008-04

Disease caused by protozoan parasites of the genus *Leishmania,* named in 1901 for British Army doctor William Leishman, who developed a stain to detect the agent*. *It is transmitted by the bite of certain species of sand fly, including the genus *Lutzomyia* in the New World and *Phlebotomus* in the Old World. 

Leishmaniasis has 2 major forms: cutaneous, characterized by skin sores, and visceral, which affects internal organs and is characterized by high fever, substantial weight loss, swelling of the spleen and liver, and anemia. If untreated, the disease is universally fatal within 2 years. Visceral leishmaniasis is also called kala-azar, a Hindi term meaning “black fever.” The causal agent, *Leishmania donovani,* was also named for physician Charles Donovan, who discovered the agent in India in 1903. An estimated 500,000 cases occur each year; India has the greatest number, followed by Bangladesh, Brazil, Nepal, and Sudan.

